# Evidence of Hearing Loss and Unrelated Toxoplasmosis in a Free-Ranging Harbour Porpoise (*Phocoena phocoena*)

**DOI:** 10.3390/ani11113058

**Published:** 2021-10-26

**Authors:** Maria Morell, Lonneke L. IJsseldijk, Alinda J. Berends, Andrea Gröne, Ursula Siebert, Stephen A. Raverty, Robert E. Shadwick, Marja J. L. Kik

**Affiliations:** 1 Institute for Terrestrial and Aquatic Wildlife Research, University of Veterinary Medicine Hannover, Foundation, 25761 Büsum, Germany; ursula.siebert@tiho-hannover.de; 2 Zoology Department, The University of British Columbia, Vancouver, BC V6T 1Z4, Canada; stephen.raverty@gov.bc.ca (S.A.R.); shadwick@zoology.ubc.ca (R.E.S.); 3 Department of Biomolecular Health Sciences, Division of Pathology, Faculty of Veterinary Medicine, Utrecht University, 3584 CL Utrecht, The Netherlands; a.j.berends@uu.nl (A.J.B.); a.grone@uu.nl (A.G.); m.kik@uu.nl (M.J.L.K.); 4 Animal Health Center, Ministry of Agriculture, Abbotsford, BC V3G 2M3, Canada

**Keywords:** *Toxoplasma gondii*, North Sea, live stranding, post-mortem examination, encephalitis, noise-induced hearing loss, inner ear, hair cell

## Abstract

**Simple Summary:**

Evidence of hearing impairment was identified in a female harbour porpoise (*Phocoena phocoena*) on the basis of inner ear analysis. The animal live stranded on the Dutch coast at Domburg in 2016 and died a few hours later. Ultrastructural examination of the inner ear revealed evidence of sensory cell loss, which is compatible with noise exposure. In addition, histopathology also revealed multifocal necrotising protozoal encephalitis. A diagnosis of toxoplasmosis was confirmed by positive staining of tissue with anti-*Toxoplasma gondii* antibodies; however, *T. gondii* tachyzoites were not observed histologically in any of the examined tissues. This is the first case of presumptive noise-induced hearing loss and demonstration of *T. gondii* cysts in the brain of a free-ranging harbour porpoise from the North Sea.

**Abstract:**

Evidence of hearing impairment was identified in a harbour porpoise (*Phocoena phocoena*) on the basis of scanning electron microscopy. In addition, based on histopathology and immunohistochemistry, there were signs of unrelated cerebral toxoplasmosis. The six-year old individual live stranded on the Dutch coast at Domburg in 2016 and died a few hours later. The most significant gross lesion was multifocal necrosis and haemorrhage of the cerebrum. Histopathology of the brain revealed extensive necrosis and haemorrhage in the cerebrum with multifocal accumulations of degenerated neutrophils, lymphocytes and macrophages, and perivascular lymphocytic cuffing. The diagnosis of cerebral toxoplasmosis was confirmed by positive staining of protozoa with anti-*Toxoplasma gondii* antibodies. Tachyzoites were not observed histologically in any of the examined tissues. Ultrastructural evaluation of the inner ear revealed evidence of scattered loss of outer hair cells in a 290 µm long segment of the apical turn of the cochlea, and in a focal region of ~ 1.5 mm from the apex of the cochlea, which was compatible with noise-induced hearing loss. This is the first case of concurrent presumptive noise-induced hearing loss and toxoplasmosis in a free-ranging harbour porpoise from the North Sea.

## 1. Introduction

The harbour porpoise (*Phocoena phocoena* Linnaeus 1758) is the most abundant cetacean species in the North-East Atlantic Ocean and adjacent North Sea, both in terms of sightings [1] and of strandings [2]. The harbour porpoise is protected under the EU Habitats Directive, and Marine Strategy Framework Directive (MSFD), Natura 2000 and the Agreement on the Conservation of Small Cetaceans of the Baltic, North East Atlantic, Irish, and North Seas (ASCOBANS), which were established to ensure the conservation of small cetacean populations in these waters [3,4]. Due to the statutory requirements of these regional and international agreements, systems for the reporting, documenting and retrieving of stranded and bycaught cetaceans have been in place in many countries bordering the North Sea [2]. Additionally, in most north-western European countries, including the Netherlands, post-mortem programs were established to study a range of threats affecting stranded individuals and population status. 

Harbour porpoises confront several anthropogenic and natural threats, including underwater noise pollution. There is an increasing concern on how man-made underwater noise exposure affects cetaceans and their hearing capabilities [5,6]. As hearing is fundamental to cetaceans, changes to their auditory capabilities may impact their ability to carry out vital activities. Previous studies have shown that the cochlea of the harbour porpoise contains two types of auditory sensory cells, the inner hair cells (IHCs) and the outer hair cells (OHCs) [7,8,9]. As in terrestrial mammals, the hair cells are arranged in one single row of IHCs and three rows of OHCs within the organ of Corti, or hearing organ. The disposition of sensory and supporting cells in the apex of the cochlea (the tip of the spiral, where the lowest frequencies are encoded) is variable. However, a recent study has described the arrangement of sensory cells in the apex of the harbour porpoise [10], providing baseline information on the common pattern in these species.

Ultrastructural alterations can be detected in the sensory cells as a result of high intensity and/or long duration sound exposure [11]. These alterations include hair cell apoptosis. When a mammalian cochlear hair cell dies, the contiguous supporting cells actively participate in hair-cell elimination, resulting in a distinct scar [12]. The presence of scars within hair cell rows can be distinguished from artefacts that may derive from autolysis and is an important criterion to assess for prior noise-induced cochlear lesions [8,13].

Frequent causes of death of harbour porpoises in the Netherlands include fisheries bycatch [14], grey seal predation [15] and a range of infectious diseases, including viral, mycotic and bacterial pathogens (e.g., [16,17,18,19]). Recently, concerns have been raised about contamination of marine aquatic life with the zoonotic, protozoal parasite *Toxoplasma gondii,* which is capable of infecting a variety of terrestrial and marine warm-blooded animals, including harbour porpoises [20,21,22]. The definitive hosts of *T. gondii* are felids. Through a sexual phase in their intestine, *T. gondii* oocysts can be introduced into the environment through contaminated faeces [23]. Oocysts can subsequently reach the sea through coastal run-off, with multiple studies demonstrating the presence of *T. gondii* in marine mammal species (reviewed in Dubey and colleagues [22]). To date, only serological evidence of *T. gondii* exposure has been reported for free-ranging harbour porpoises from the North-East Atlantic and adjacent waters [21,24,25].

Understanding of natural and anthropogenic causes of mortality in stranded cetaceans is vital for evaluating marine mammal and ecosystem health and species conservation and sustainability [26]. Post-mortem examinations of stranded small cetaceans and the diagnosis of (emerging) infectious diseases, such as toxoplasmosis, as well as anthropogenic threats, such as noise-induced hearing loss, contribute to quantifying the health status and conservation of harbour porpoises inhabiting the southern North Sea. Herein we describe an extensive investigation of a live stranded harbour porpoise found on the coast of the Netherlands, including pathological investigations, molecular screening for pathogens, life history data, toxicology and inner ear analyses, to determine a cause of death and ante-mortem health status of the animal.

## 2. Materials and Methods

### 2.1. Stranding and Necropsy

An adult live stranded female harbour porpoise (UT1535) was recovered on the beach of the town of Domburg on the Dutch North Sea coast in July 2016 and died en route to a regional rehabilitation centre. In the Netherlands, necropsies of marine mammals are conducted at the division of Pathology, Department of Biomolecular Health Sciences of the Faculty of Veterinary Medicine of Utrecht University (UU) following an internationally standardised protocol [27]. The harbour porpoise was transported to UU directly after death and the necropsy started 3.5 h post mortem. The animal was photographed and weighed, and its length, girth and blubber thickness immediately anteriorly to the dorsal fin were measured and recorded [27].

All organs were grossly examined and, samples were systemically collected for histopathology, including skin, skeletal muscle, lung, heart, thymus, thyroid, stomachs, pancreas, spleen, liver, adrenals, kidney, intestine, urinary bladder, reproductive organs, mammary gland, eyes, brain and spinal cord, and various lymph nodes. This suite of tissues and representative samples of gross lesions were preserved in 10% neutral buffered formalin, processed by conventional histologic techniques, embedded in paraffin, sectioned at 4 µm and stained with haematoxylin and eosin (HE). Additional recuts and special stains of the cerebrum tissue, included periodic acid–Schiff (PAS) stain, to assess the presence of fungal organisms, and Ziehl–Neelsen (ZN) stain, to assess the presence of acid-fast mycobacteria. Sections of the cerebral lesions were stained immunohistochemically with polyclonal rabbit antibody against *T. gondii* (LSBio, LS C312239, 1:200) following a standard avidin–biotin complex protocol [28]. Control sections were processed without primary antibodies. A frozen blood sample was examined by an in-house immunoblot method using *T. gondii* surface antigen p30 (SAG1) as described previously [29] with the modification that, instead of the peroxidase-conjugated anti-mouse IgG, a peroxidase-conjugated Protein A/G (Pierce™ Recombinant Protein A/G, Peroxidase Conjugated; ThermoFisher Scientific) was used.

### 2.2. Inner Ear Analysis

The inner ears of the harbour porpoise were collected and fixed within 4 h post mortem following earlier published methods [30]. The ears were shipped to the University of British Columbia (UBC), Canada, for analysis (with CITES export permit number 16NL234380/12). The right inner ear was processed for immunofluorescence (IF) and the left inner ear for scanning electron microscopy (SEM), following previously optimised protocols (see Morell and colleagues [13] for SEM and Morell and colleagues [9,31] for IF).

#### 2.2.1. Right Inner Ear: Immunofluorescence (IF)

The right periotic bone was decalcified with 14% EDTA (ethylenediaminetetraacetic acid, Sigma-Aldrich, St. Luis, MO, USA) tetrasodium salt (pH 7.4) for 43 days at room temperature. Then, the bone was removed, and the cochlea dissected using the whole-mount technique. The OHCs of the organ of Corti were labelled with anti-prestin antibody (Santa Cruz Biotechnology, Inc., Dallas, TX, USA, SC22692, 1:200), the IHCs and OHCs with anti-myosin VI antibody (Proteus Biosciences Inc., Waltham, MA, USA, 256791, 1:500), and type I afferent innervation was labelled with anti-neurofilament 200 kD antibody (Sigma-Aldrich, St. Luis, MO, USA, N0142, 1:400). Nuclei were counterstained with DAPI (4′, 6-diamidino-2′-phenylindole, dihydrochloride; Thermo Scientific, Rockford, lL, USA, 62247, 1:1000) and with the secondary antibodies (Alexa Fluor^®^ 488 donkey anti-goat IgG, Alexa Fluor^®^ 568 donkey anti-rabbit IgG, Alexa Fluor^®^ 647 donkey anti-mouse IgG; Molecular Probes, Inc. Eugene, OR, USA A11055, A10042 and A31571, respectively; 1:400). Three small sub-segments were processed as controls: (1) control for the specificity of binding by the primary antibody (the sub-segment was incubated with normal IgG at the same concentration at which the primary antibody was used and then incubated with the same concentrations of the secondary antibody and DAPI as used on experimental segments); (2) control for non-specific binding of the secondary antibodies (the sub-segment was incubated without the primary antibodies, but with the same concentrations of the secondary antibody and DAPI as used on experimental segments) and (3) control for autofluorescence (no primary and no secondary antibodies were used).

The right inner ear was evaluated using an Olympus FV1000 confocal microscope at the UBC Bioimaging Facility. Micrographs of the three controls were taken using the same settings as their respective treatments (i.e., same magnification and same intensity of the four lasers). Brightness and contrast were enhanced, using identical values for treatments and the respective controls.

#### 2.2.2. Left Inner Ear: Scanning Electron Microscopy (SEM)

The left periotic bone was decalcified with 14% EDTA (see above) for 37 days. The cochlea was dissected, dehydrated with increasing concentrations of ethanol, critical-point-dried with CO_2_ and coated with platinum/palladium. The left cochlea was observed using an S-4700 SEM at the UBC Bioimaging Facility.

### 2.3. Life History

The ovaries were fixed and assessed for corpora scars (following Murphy and colleagues [32,33]) with results presented by van den Heuvel-Greve and colleagues [34]. In short, ovaries were initially examined macroscopically, then serially sectioned at 0.5–2 mm slices and examined under a binocular microscope. Total numbers of ovarian corpora scars were counted, representing the number of ovulations. During the necropsy, pregnancy and lactation were confirmed by detection of a foetus and milk secretions from the mammary glands. Age was determined by counting growth layer groups in the dentine of tooth sections, using a binocular microscope following earlier published methods [35].

### 2.4. Molecular Studies

Targeted pathogen screening was conducted by PCR for *Brucella spp.*, herpesvirus, morbillivirus, *Neospora caninum* and *T. gondii*. For *Brucella spp.*, DNA was extracted from fresh lung, pulmonary lymph node and reproductive tract lymph node tissue, and for *T. gondii* and *N. caninum* from frozen as well as paraffin-embedded and fixed lung and cerebrum tissue using the DNeasy Blood and Tissue Kit (QIAGEN, Hilden, Germany) and QIAamp DNA FFPE Tissue Kit at Laboklin, according to the manufacturer’s protocol, respectively. An ADIAVET™ TOXO FAST TIME Kit von ADIAGENE Bio-X Diagnostics, according to manufacturer’s instructions, was used for the detection of *T. gondii*, while an in-house-developed PCR modified after Pereira and colleagues [36] was used for the detection of *N. caninum.* Isolated DNA was screened by real-time PCR targeting the IS711 sequences of *Brucella spp*. following Maio and colleagues [16] and by qPCR following Elmore and colleagues [37]. For herpesvirus detection, DNA was extracted from frozen lung and cerebrum tissue using the DNeasy Blood (QIAGEN) according to the manufacturer’s protocol. A nested pan-herpes PCR targeting the polymerase gene was performed as described previously [38]. For morbillivirus detection, RNA was extracted from frozen lung and cerebrum using the RNeasy mini-Kit (QIAGEN) according to the manufacturer’s protocol. A morbilli PCR targeting conserved sequences in the phosphoprotein gene was performed as described previously [39].

### 2.5. Toxicology

Blubber and milk samples were analysed for polychlorinated biphenyls (PCBs), as part of a separate study [34]. Air-exposed parts of the blubber sample were removed and the remaining sample was homogenised. Total extractable lipid levels were determined in milk and blubber and samples analysed to quantify PCBs using accelerated solvent extraction and gas chromatography coupled to a mass spectrometry (GC-MS) method. ∑17PCB is given, based on the most relevant PCB congeners (congener # 47, 49, 52, 101, 105, 118, 128, 138, 149, 151, 153, 156, 170, 180, 187, 194 and 202). For full details, see van den Heuvel-Greve and colleagues [34].

### 2.6. Image Processing

The brightness and contrast of images were adjusted in Adobe (San Jose, CA, USA) Photoshop CC 2018.

## 3. Results

### 3.1. Pathological Findings

**Figure 1:**
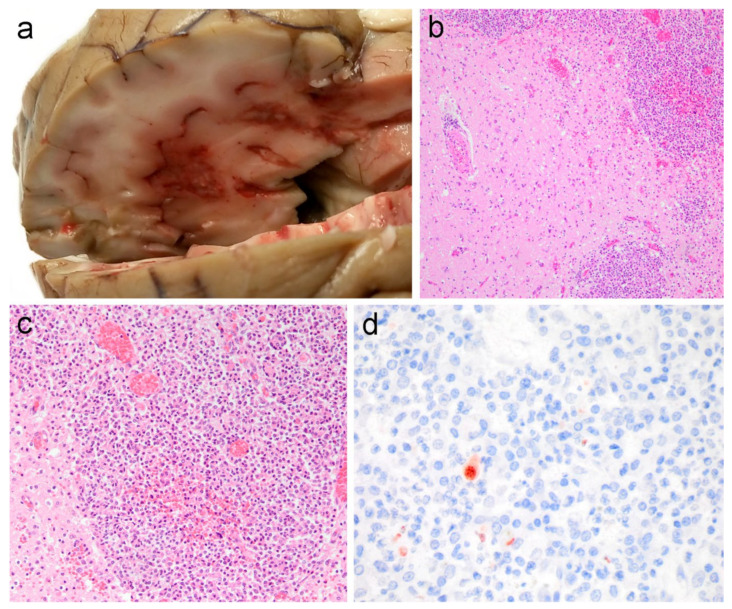
(**a**) Gross dissection of the cerebrum partially fixed in 10% neutral buffered formalin showing the extent and location of the lesions macroscopically. (**b**,**c**) Cerebrum (HE ×10 in (**b**) and HE ×20 in (**c**)) extensive mixed inflammatory reaction of degenerated neutrophils, macrophages and lymphocytes. (**d**) Immunopositivity for *Toxoplasma gondii* (×60). The background of the image in panel (**a**) was removed.

**Figure 2:**
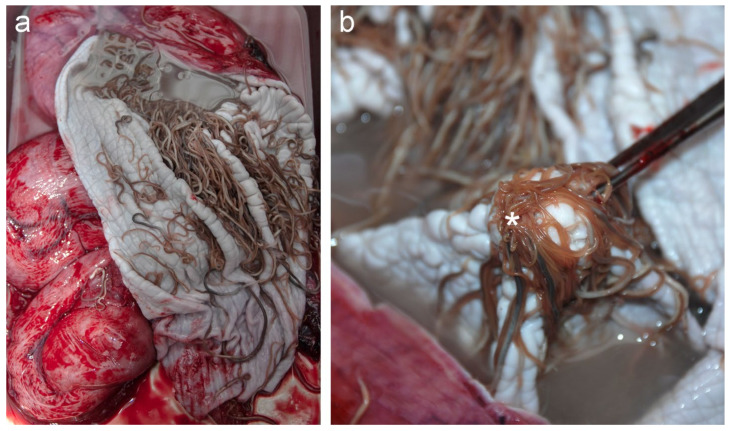
(**a**) Nematode infestation of the forestomach, with (**b**) focal nodular hyperkeratosis and hyperplasia with central ulceration (crateriform) of the mucosa (asterisk), which is consistently observed in gastric mucosa of the first stomach compartment in animals infected with these nematodes.

At necropsy, the adult female measured 146 cm total body length and weighed 46 kg. Based on the absence of visceral fat, muscle atrophy and blubber thicknesses of 6–8 mm she was in poor nutritional condition. Macroscopic lesions included multiple foci of necrosis in the cerebrum (Figure 1a). Additionally, the animal had verminous bronchopneumonia with a moderate number of nematodes (*Pseudaliidae*) in both the bronchi and in the lumen of multiple pulmonary vessels. There were large numbers of nematodes morphologically consistent with *Anisakis simplex* present in the lumen of the forestomach, with multifocal mucosal ulceration and numerous invading nematodes (Figure 2). A moderate amount of opaque white fluid was in the forestomach with a distinct acetone-smell, suggestive of ketosis. There was no ingesta observed within the gastrointestinal tract. Nematodes, morphologically consistent with *Stenurus minor*, were bilaterally present in large numbers in cranial sinuses (peribullar and pterygoid) and in the tympanic cavities. A small number of trematodes, morphologically consistent with *Campula oblonga*, were observed in the hepatobiliary arcade.

Histopathology of the cerebrum revealed extensive necrosis with haemorrhage and degenerated neutrophils, lymphocytes and macrophages (Figure 1b–d). There was lymphocytic perivascular cuffing in the cerebral white and grey matter and in the meninges. Additional recuts and special stains of the cerebrum for fungi and acid-fast bacilli were negative. In the cerebellum, there were scattered foci of acute haemorrhage. In multiple regions of the lung, there was diffuse, moderate to severe interstitial pneumonia, and alveolar spaces were filled with eosinophils, lymphocytes and foamy macrophages. There was a mild multifocal granulomatous and eosinophilic cholangiohepatitis with multifocal hepatocellular hemosiderosis. The forestomach featured multifocal, severe necroerosive and ulcerative nonsuppurative gastritis with numerous foamy macrophages and occasionally, cross-sections of adult nematodes. There was white pulp hyperplasia and hemosiderin-laden macrophages throughout the splenic stroma with multisystemic congestion, including the eyes, thyroid, several lymph nodes, spleen, adrenal and kidney. No lesions were detected in any of the other examined tissues.

### 3.2. Immunohistochemistry and Molecular Studies

The presence of *Toxoplasma gondii* cysts in the cerebrum was confirmed by positive staining with anti-*T. gondii* antibodies (Figure 1) and no tachyzoites were observed histologically in any of the organs. All additional ancillary diagnostic studies to screen for recognised pathogens were negative. No nonspecific binding was noted in control sections.

### 3.3. Inner Ear Analyses

#### 3.3.1. Right Inner Ear: IF

**Figure 3:**
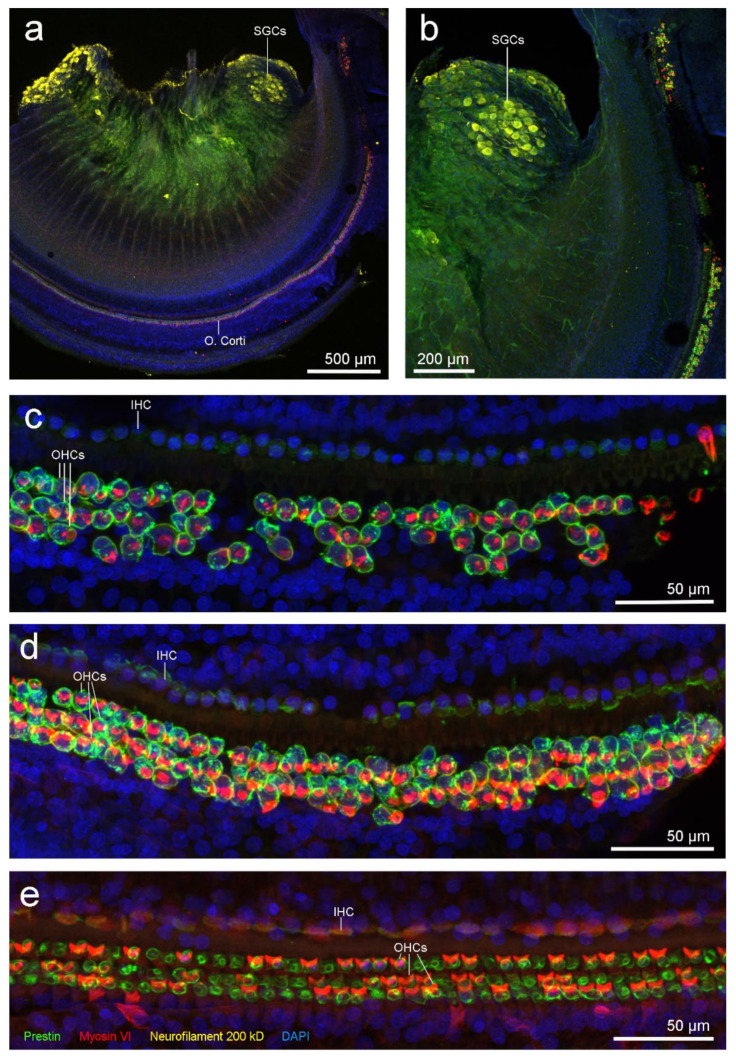
Immunofluorescence micrographs of the right cochlea from individual UT1535, labelled with anti-prestin (green), anti-myosin VI (red) and anti-neurofilament 200 kD (yellow) antibodies and DAPI (blue). (**a**–**d**) Apical turn; note the missing outer hair cells (OHCs) in (**c**) and a few OHCs missing from the third row in (**d**). (**e**) Organ of Corti of the lower apical turn. All these micrographs are z-projections from confocal images, whose slice thicknesses were 75 µm (**a**), 10 µm (**b**) and 1.5 µm (**c**–**e**). IHC, inner hair cells; O. Corti, organ of Corti; SGCs, spiral ganglion cells.

All three control treatments for nonspecific immunofluorescence were negative. Anti-neurofilament 200 kD antibody had penetration problems (Figure 3a,b) since the Rosenthal’s canal is very thick in toothed whales. The Rosenthal’s canal is the region where the spiral ganglion cells (i.e., afferent nerve cell bodies) are located. However, since spiral ganglion cells are very autofluorescent, they were observed throughout the cochlear spiral and there was no visible neuronal degeneration.

Hair cells of the organ of Corti were present through the spiral except in the most apical portion. There were some missing OHCs in the first 200 µm from the apex, possibly due to normal individual apex variability. There was no information in the adjoining 600 µm segment due to a dissection or processing artefact. From 0.8 to 1 mm from the apex, the next 200 µm, had missing OHCs from the three rows (Figure 3c). Then, there was a 500 µm region with scattered OHCs loss (Figure 3d), and thereafter, three intact rows of OHCs were present consistently all along the spiral (Figure 3e).

#### 3.3.2. Left Inner Ear: SEM

**Figure 4:**
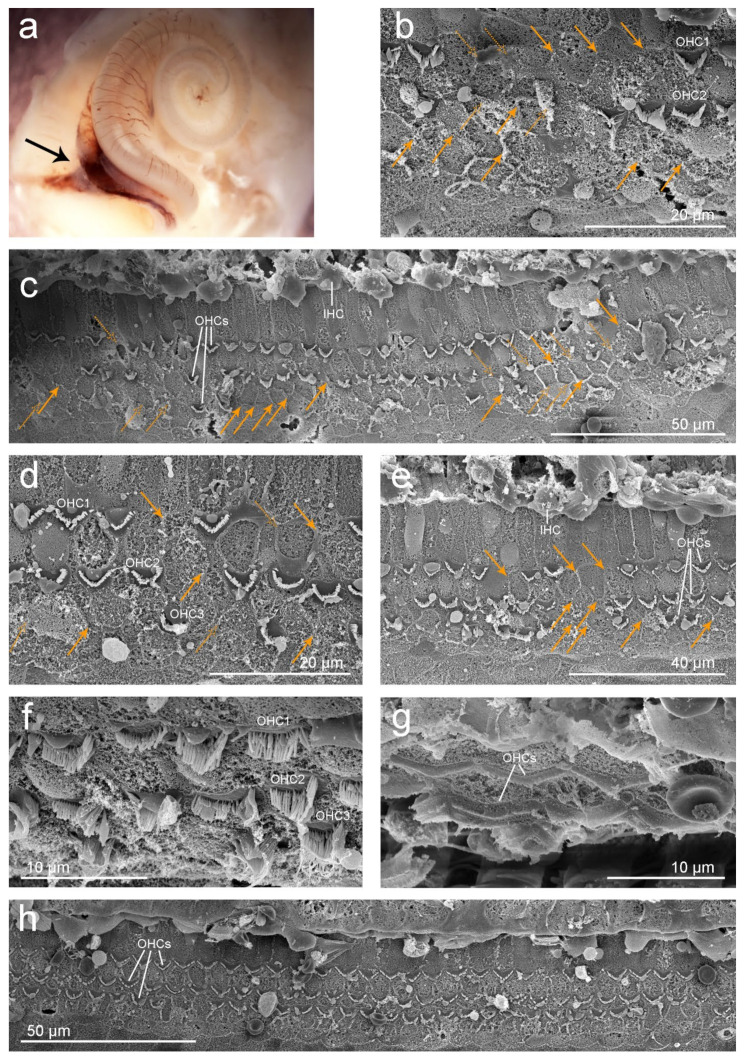
(**a**) Subgross image of the left cochlea from individual UT 1535. The black arrow highlights focal to segmental congestion involving the vein at the cochlear aqueduct. (**b**–**h**) Scanning electron microscope images. (**b**–**e**) Evidence of outer hair cell (OHC) death at the upper apical turn, highlighted with orange arrows. The dashed orange arrows indicate potential evidence of lesions. Panel (**c**) is located ~525 µm from the beginning of the apex. The micrograph in panel (**b**) is taken 15 µm after that of panel (**c**), towards the base. Panels (**d**) and (**e**) are located ~1.5 mm from the beginning of the apex. Organ of Corti with three rows of OHCs and one row of inner hair cells (IHCs) of the upper apical turn (**f**), upper (**h**) and lower (**g**) basal turn.

Examination by a dissecting microscope revealed focal to segmental congestion involving the vein towards the cochlear aqueduct (black arrow in Figure 4a).

Ultrastructural evaluation with SEM revealed that the organ of Corti was absent in the first 525 µm segment from the apex and moderately well preserved through the rest of the spiral turn. In the first region where the organ of Corti cells were present, there were scars as a result of OHC death in a 290 µm segment (Figure 4b,c) and in a focal area approximately 1.5 mm from the apex (Figure 4d,e). Orange arrows in Figure 4 highlight the position of the scars and dashed arrows mark the potential remains of OHCs or evidence of scars.

Three rows of OHCs and one row of IHCs could be identified in the rest of the cochlear spiral (Figure 4f–h). Focal mild haemorrhage was observed in the vestibular scala of the lower basal turn.

### 3.4. Life History

Upon necropsy, the left uterine horn was distended and there was endometrial oedema and congestion with a prominent luteal body on the left ovary. These findings were consistent with recent gestation. In addition, the mammary glands were secretory with thick, white contents. No calf had been observed during the live stranding nor in the surroundings of the stranding location. Twelve corpora scars, including one corpus luteum, were counted in the left ovary and none were observed in the right. Tooth examination revealed a 6-year-old female (results taken from van den Heuvel-Greve and colleagues [34]).

### 3.5. Toxicology

The ∑17PCB in blubber was 5.1 mg/kg lipid weight (lw) and 16.8 mg/kg lw in milk, indicative of active offloading through lactation (for details, see van den Heuvel-Greve and colleagues [34]).

## 4. Discussion

Inner ear analysis revealed evidence of OHC death in the apical turn of the cochlea, which in mammals results in permanent hearing loss [40,41,42]. Specifically, evaluation of the left cochlea by SEM showed scattered OHC loss from 525–815 µm and focally at 1.5 mm from the apex. The lack of OHCs (especially OHCs from the third row) can be considered part of the normal apical variability in harbour porpoise and other animals [10]. However, because the ratio of hair cells and supporting Deiters cells is 1:1 [10], there was strong evidence of scars as a result of OHC death by apoptosis (orange arrows in Figure 4) in this case, rather than artefact or normal anatomic variation. The focal mild haemorrhage observed in the vestibular scala of the lower basal turn was possibly an artefactual transfer of erythrocytes from around the vein towards the cochlear aqueduct that may have occurred during the dissection or critical point drying process. The lack of positive staining in OHCs from the right apical turn (Figure 3b,c) could also have been due to hair cell autolysis, and not necessarily a consequence of OHC death. The selection of antibodies used for this sample was optimal to discriminate between newly formed lesions and old ones [9]. In the case of recent noise-induced hearing loss, prestin clumps in the cytoplasm of the supporting cells occurs in guinea pigs up to 9–10 days post-exposure [43]. If a similar rate of scar formation occurs in harbour porpoises, the missing hair cells from the right ear of this animal were not associated with recent acoustic injury.

Permanent hearing loss can be caused by several factors, including exposure to noise, ototoxic drugs, or PCBs, age, congenital or immunological disorders, and other infections [44,45,46,47,48,49]. Congenital toxoplasmosis can cause sensorineural hearing loss in humans [50], but little is known on the specific characteristics of the type and level of hearing loss, or of other disorders possibly involved in the auditory processing [51]. Histopathological and immunohistochemical studies in infants with congenital toxoplasmosis showed a decreased neuron population and in one individual a significant loss of OHCs, especially severe at the basal turn [52]. However, in this case, it is highly unlikely that the toxoplasmosis and the lesions found in the inner ear in our study are related. There were no apparent degenerate spiral ganglion neurons, nor OHC loss in the base of the cochlea. In humans and terrestrial mammals, depending on the aetiology, pathogenic impacts of sensorineural hearing loss may either affect the entire hearing range uniformly, or result in hearing loss predominantly in the high frequencies [53,54,55,56] encoded at the cochlear base. To the best of our knowledge, at present there are no recognised pathogens exclusively and focally affecting the apex of the cochlea. Therefore, based on the location and pattern of OHC loss in this case, an infectious aetiology is unlikely. This individual was a relatively young adult (six years old) and it is unlikely that the ultrastructural results were related to presbycusis, since age-related hearing loss primarily starts to affect the high frequencies [44]. Moreover, auditory evoked potential studies in cetacean species have demonstrated that aged individuals typically present high-frequency hearing loss [57,58]. In addition, experiments in terrestrial mammals show that hair cell loss and spiral ganglion cell degeneration typically affect initially and more severely the base of the cochlea in cases of barotrauma, or exposure to ototoxic drugs (e.g., gentamicin or amikacin) [59,60]. No medication was administrated to this animal after stranding. There were no apparent lesions in the sensorineural epithelium at the base of the cochlea that were consistent with presbycusis, barotrauma or ototoxic drug exposure. Studies in rats showed that developmental exposure to PCBs can result in severe hearing loss with corresponding mild to moderate loss of OHCs in the upper-middle and apical turns [47,48,49]. This animal had a total concentration of 5.1 mg/kg ∑PCBs (lw) in her blubber and 16.8 mg/kg (lw) in her milk [34]. Levels in blubber were below the threshold considered to cause physiological effects [61,62]. This makes it unlikely that overexposure to PCBs was a plausible cause for the OHC death, although more research is needed on PCB levels in different types of tissues and fluids and their effects on hearing. Other, rarely reported causes in humans, such as developmental defects or immunological disorders, have not yet been described in marine mammals and were deemed unlikely for their extremely low prevalence (less than 1% of all cases of hearing impairment in humans [63]).

The type of lesions seen in the cochlea in our study are compatible with noise-induced hearing loss. The location of noise-induced lesions within the cochlea depends on the frequency of the source. Studies on noise-induced hearing loss in terrestrial and marine mammals exposed to high intensity noise levels showed that the frequency of maximum hearing loss was a half octave, up to one octave, above the exposing tone [64,65,66,67]. Future research on predicting the cochlear frequency maps (i.e., frequency distribution along the cochlear spiral) based on morphological features on harbour porpoises is needed. These maps are important for determining the frequency range that is impaired if lesions are found. In addition, in cases of noise-induced hearing loss, frequency maps can ultimately provide key information on the frequency characteristics of the causal sources of the cochlear lesions.

This animal live stranded on the Dutch coast in 2016 and the most significant gross findings were multifocal necrosis and haemorrhage of the cerebrum and generalised emaciation. Positive staining of cerebrum tissue with anti-*T. gondii* antibodies confirmed protozoal encephalitis due to *T. gondii* infection while herpesvirus, morbillivirus and *N. caninum* were negative. 

*Toxoplasma gondii* is generally considered a sporadic infection of aquatic and marine mammals [68,69]. Exposure to *T. gondii* is likely from terrestrial run-off and infection may be predisposed or possibly exacerbated by immunosuppressive, or debilitating factors, such as chemical contamination by PCBs, pregnancy, malnutrition, morbillivirus infection and other processes [21,70,71,72,73,74]. Harbour porpoises have a coastal distribution and, in this case, parasite exposure was most likely via terrestrially sourced faecal oocysts with possible bioaccumulation in prey species. There was another porpoise diagnosed with a fatal disseminated *T. gondii* infection. This animal was born and held in a semi-open outdoor facility [20] and faecal contamination of rainwater was deemed the route of infection. For pelagic cetacean species, such as striped dolphin (*Stenella coeruleoalba*), reports of toxoplasmosis are more common [69] and an “open sea *T. gondii* life cycle” with more virulent genotypes has been suggested, but not yet confirmed [75,76]. These cases highlight the need for more comparative studies on protozoan genotyping and host factors involved in recruitment and infection [75,77].

Severe and multiple-organ metazoan parasitic infections were also detected in this porpoise. The degree of emaciation and parasitic burden in this animal indicates generalised debilitation and impaired health status. However, in harbour porpoises from the southern North Sea, severity of parasite infections increased with total length of the host and all examined adults presented multisystemic parasitism [78]. The lungs typically had high parasitic loads, with associated bronchopneumonia and vasculitis [79,80,81,82]. A degree of immunosuppression during pregnancy is also recognised in terrestrial mammals and if this phenomenon occurs in cetaceans, may also have contributed to the impaired health status of this animal. Studies on cetaceans have shown that individuals with an impaired immune system have a higher susceptibility to infectious diseases [83,84]. There were no discernible viral inclusions in the examined tissue sections and molecular studies proved negative of morbillivirus. The blubber contaminant levels were below the thresholds reported for adverse effects [33,85], although offloading through lactation was apparent [34].

Harbour porpoises use very high frequency acoustic signals of relative narrow bandwidth, with a frequency peak of ~130 kHz and the main energy between 110 and 150 kHz [86,87,88]. However, behavioural audiograms show that harbour porpoises can hear from 125 Hz to 180 kHz with a maximum of hearing sensitivity of 100–125 kHz [89]. The relevance and impact of having a hearing impairment in the lower frequencies in harbour porpoise is not clear. However, it is possible that a hearing impairment could have made this individual more vulnerable to other threats. The effects of noise pollution on cetaceans are not limited to damage to the inner ear and can include a range of physiological and pathological changes, which may induce adverse behavioural responses [90]. The detection and quantification of the effects of noise pollution is highly challenging in free-ranging cetaceans. With the diagnostic investigation into this porpoise, we show that age, toxoplasmosis, barotrauma, potential previous ototoxic drug exposure and PCB pollution were unlikely the cause of hearing loss. The location and focal distribution of the scars in the cochlea suggest that exposure to anthropogenic sound was the most likely cause of the lesions. There is an urgent need to increase our understanding of the consequences of anthropogenic sound exposure on marine mammal hearing. Continued comprehensive diagnostic research programs on fresh, stranded cetaceans is highly recommended. These investigations increase our knowledge with the auditory systems of these species and ultimately to help distinguishing between changes attributable to noise pollution, infectious or other causes of hearing loss.

## 5. Conclusions

This study presents a case with evidence of hearing loss, which was compatible with noise exposure. In addition, histopathological and immunohistochemical analysis revealed a case of toxoplasmosis in a wild harbour porpoise stranded in the North Sea with severe lesions in the brain.

## Data Availability

The original contributions presented in the study are included in the article. Further inquiries can be directed to the corresponding authors.

## References

[1] Hammond P.S., Berggren P., Benke H., Borchers D.L., Collet A., Heide-Jørgensen M.P., Heimlich S., Hiby A.R., Leopold M.F., Øien N. (2002). Abundance of harbour porpoise and other cetaceans in the North Sea and adjacent waters. J. Appl. Ecol..

[2] IJsseldijk L.L., ten Doeschate M.T., Brownlow A., Davison N.J., Deaville R., Galatius A., Haelters J., Jepson P.D., Keijl G.O., Kinze C.C. (2020). Spatiotemporal mortality and demographic trends in a small cetacean: Strandings to inform conservation management. Biol. Conserv..

[3] Green M., Caddell R., Eisfeld S., Dolman S., Simmonds M. (2012). Looking Forward to ‘Strict Protection’: A Critical Review of the Current Legal Regime for Cetaceans in UK Waters.

[4] Camphuysen C.J., Siemensma M.L. (2011). Conservation Plan for the Harbour Porpoise Phocoena phocoena in the Netherlands: Towards a Favourable Conservation Status.

[5] Erbe C., Dunlop R., Dolman S., Slabbekoorn H., Dooling R.J., Popper A.N., Fay R.R. (2018). Effects of Noise on Marine Mammals. Effects of Anthropogenic Noise on Animals.

[6] Simmonds M.P., Dolman S.J., Jasny M., Parsons E.C.M., Weilgart L., Wright A.J., Leaper R. (2014). Marine noise pollution—Increasing recognition but need for more practical action. J. Ocean Technol..

[7] Morell M. (2012). Ultrastructural Analysis of Odontocete Cochlea. Ph.D. Thesis.

[8] Morell M., Lenoir M., Shadwick R.E., Jauniaux T., Dabin W., Begeman L., Ferreira M., Maestre I., Degollada E., Hernandez-Milian G. (2015). Ultrastructure of the odontocete organ of Corti: Scanning and transmission electron microscopy. J. Comp. Neurol..

[9] Morell M., Vogl W., Ijsseldijk L.L., Piscitelli-Doshkov M., Tong L., Ostertag S., Ferreira M., Fraija-Fernandez N., Colegrove K., Puel J.L. (2020). Echolocating whales and bats express the motor protein prestin in the inner ear: A potential marker for hearing loss. Front. Vet. Sci..

[10] Morell M., IJsseldijk L.L., Piscitelli-Doshkov M., Ostertag S., Estrade V., Haulena M., Doshkov P., Bourien J., Raverty S.A., Siebert U. (2021). Cochlear apical morphology in toothed whales: Using the pairing hair cell—Deiters’ cell as a marker to detect lesions. Anat. Rec..

[11] Bredberg G., Ades H.W., Engstrom H. (1972). Scanning electron microscopy of normal and pathologically altered organ of Corti. Acta Otolaryngol..

[12] Raphael Y., Altschuler R.A. (1991). Reorganization of cytoskeletal and junctional proteins during cochlear hair cell degeneration. Cell Motil. Cytoskeleton.

[13] Morell M., Brownlow A., McGovern B., Raverty S.A., Shadwick R.E., André M. (2017). Implementation of a method to visualize noise-induced hearing loss in mass stranded cetaceans. Sci. Rep..

[14] IJsseldijk L.L., Scheidat M., Siemensma M.L., Couperus B., Leopold M.F., Morell M., Gröne A., Kik M.J.L. (2021). Challenges in the assessment of bycatch: Postmortem findings in harbor porpoises (*Phocoena phocoena*) retrieved from gillnets. Vet. Pathol..

[15] Leopold M.F., Begeman L., van Bleijswijk J.D., IJsseldijk L.L., Witte H.J., Gröne A. (2015). Exposing the grey seal as a major predator of harbour porpoises. Proc. R. Soc. B.

[16] Maio E., Begeman L., Bisselink Y., van Tulden P., Wiersma L., Hiemstra S., Ruuls R., Gröne A., Roest H.I.J., Willemsen P.T.J. (2014). Identification and typing of *Brucella spp.* in stranded harbour porpoises (*Phocoena phocoena*) on the Dutch coast. Vet. Microbiol..

[17] van Beurden S.J., IJsseldijk L.L., Ordonez S.R., Förster C., de Vrieze G., Gröne A., Verheije M.H., Kik M. (2015). Identification of a novel gammaherpesvirus associated with (muco) cutaneous lesions in harbour porpoises (*Phocoena phocoena*). Arch. Virol..

[18] Foster G., Whatmore A.M., Dagleish M.P., Malnick H., Gilbert M.J., Begeman L., Macgregor S.K., Davison N.J., Roest H.J., Jepson P. (2019). Forensic microbiology reveals that *Neisseria animaloris* infections in harbour porpoises follow traumatic injuries by grey seals. Sci. Rep..

[19] Kapetanou A., IJsseldijk L.L., Willems D.S., Broens E.M., Everaarts E., Buil J.B., Verweij P.E., Kik M.J.L., Gröne A. (2020). Mycotic infections in free-ranging harbor porpoises (*Phocoena phocoena*). Front. Mar. Sci..

[20] Herder V., van de Velde N., Kristensen J.H., Van Elk C., Peters M., Kilwinski J., Schares G., Siebert U., Wohlsein P. (2015). Fatal disseminated *Toxoplasma gondii* infection in a captive harbour porpoise *(Phocoena phocoena*). J. Comp. Pathol..

[21] van de Velde N., Devleesschauwer B., Leopold M., Begeman L., IJsseldijk L.L., Hiemstra S., Jzer J., Brownlow A., Davison N., Haelters J. (2016). *Toxoplasma gondii* in stranded marine mammals from the North Sea and Eastern Atlantic Ocean: Findings and diagnostic difficulties. Vet. Parasitol..

[22] Dubey J.P., Murata F.H., Cerqueira-Cézar C.K., Kwok O.C., Grigg M.E. (2020). Recent epidemiologic and clinical importance of *Toxoplasma gondii* infections in marine mammals: 2009–2020. Vet. Parasitol..

[23] Dubey J.P. (2010). Toxoplasmosis of Animals and Humans.

[24] Cabezón O., Resendes A.R., Domingo M., Raga J.A., Agustí C., Alegre F., Mons J.L., Dubey J.P., Almería S. (2004). Seroprevalence of *Toxoplasma gondii* antibodies in wild dolphins from the Spanish Mediterranean coast. J. Parasitol..

[25] Forman D., West N., Francis J., Guy E. (2009). The sero-prevalence of *Toxoplasma gondii* in British marine mammals. Meml. Inst. Oswaldo Cruz.

[26] Bossart G.D. (2011). Marine mammals as sentinel species for oceans and human health. Vet. Pathol..

[27] IJsseldijk L.L., Brownlow A.C., Mazzariol S. (2019). Best Practice on Cetacean Post Mortem Investigation and Tissue Sampling. Joint ACCOBAMS/ASCOBANS Document. https://osf.io/zh4ra/.

[28] Key M., Kumar G.L., Rudbeck L. (2009). Immunohistochemical Staining Methods. Immunohistochemical Staining Methods.

[29] Burrells A., Taroda A., Opsteegh M., Schares G., Benavides J., Dam-Deisz C., Bartley P.M., Chianini F., Villena I., van der Giessen J. (2018). Detection and dissemination of *Toxoplasma gondii* in experimentally infected calves, a single test does not tell the whole story. Parasites Vectors.

[30] Morell M., André M. (2009). Cetacean Ear Extraction and Fixation Protocol. http://www.zoology.ubc.ca/files/Ear_extraction_and_fixation_protocol_UBC.pdf.

[31] Morell M., Raverty S.A., Mulsow J., Haulena M., Barret-Lennard L., Nordstrom C., Venail F., Shadwick R.E. (2020). Combining cochlear analysis and auditory evoked potentials in a beluga whale with high-frequency hearing loss. Front. Vet. Sci..

[32] Murphy S., Pierce G.J., Law R.J., Bersuder P., Jepson P.D., Learmonth J.A., Addink M., Dabin W., Santos M.B., Deaville R. (2010). Assessing the effect of persistent organic pollutants on reproductive activity in common dolphins and harbour porpoises. J. Northwest Atl. Fish Sci..

[33] Murphy S., Barber J.L., Learmonth J.A., Read F.L., Deaville R., Perkins M.W., Brownlow A., Davison N., Penrose R., Pierce G.J. (2015). Reproductive failure in UK harbour porpoises *Phocoena phocoena*: Legacy of pollutant exposure?. PLoS ONE.

[34] van den Heuvel-Greve M., van den Brink A.M., Kotterman M., Kwadijk C., Geelhoed S.C.V., Murphy S., van den Broek J., Heesterbeek H., Gröne A., IJsseldijk L.L. (2021). Polluted porpoises: Generational transfer of contaminants in harbour porpoises from the southern North Sea. Sci. Total Environ..

[35] Hohn A.A., Lockyer C. (1995). Protocol for Obtaining Age Estimates from Harbour Porpoise Teeth. Biology of Phocoenids. Appendix 3, Report of the Harbour Porpoise Age Determination Workshop.

[36] Pereira G.R., Vogel F.S., Bohrer R.C., da Nóbrega J.E.J., Ilha G.F., da Rosa P.R., Glanzner W.G., Camillo G., Braunig P., de Oliveira J.F. (2014). *Neospora caninum* DNA detection by TaqMan real-time PCR assay in experimentally infected pregnant heifers. Vet. Parasitol..

[37] Elmore S.A., Jones J.L., Conrad P.A., Patton S., Lindsay D.S., Dubey J.P. (2010). *Toxoplasma gondii*: Epidemiology, feline clinical aspects, and prevention. Trends Parasitol..

[38] Van Devanter D.R., Warrener P., Bennett L., Schultz E.R., Coulter S., Garber R.L., Rose T.M. (1996). Detection and analysis of diverse herpesviral species by consensus primer PCR. J. Clin. Microbiol..

[39] Jensen T., Dietz H.H., Andersen T.H., Hammer A., Kuiken T., Osterhaus A., van de Bildt M. (2002). Another phocine distemper outbreak in Europe. Science.

[40] Saunders J.C., Cohen Y.E., Szymko Y.M. (1991). The structural and functional consequences of acoustic injury in the cochlea and peripheral auditory system: A five year update. J. Acoust. Soc. Am..

[41] Kujawa S.G., Liberman M.C. (2019). Translating animal models to human therapeutics in noise-induced and age-related hearing loss. Hear. Res..

[42] Burton J.A., Mackey C.A., MacDonald K.S., Hackett T.A., Ramachandran R. (2020). Changes in audiometric threshold and frequency selectivity correlate with cochlear histopathology in macaque monkeys with permanent noise-induced hearing loss. Hear. Res..

[43] Abrashkin K.A., Izumikawa M., Miyazawa T., Wang C., Crumling M.A., Swiderski D.L., Beyer L.A., Gong T.L., Raphael Y. (2006). The fate of outer hair cells after acoustic or ototoxic insults. Hear. Res..

[44] Johnsson L.G., Hawkins J.E. (1972). Sensory and neural degeneration with aging, as seen in microdissections of human inner ear. Ann. Otol. Rhinol. Laryngol..

[45] Black R.E., Lau W.K., Weinstein R.J., Young L.S., Hewitt W.L. (1976). Ototoxicity of amikacin. Antimicrob. Agents Chemother..

[46] Lim D.J., Dunn D.E. (1979). Anatomic correlates of noise induced hearing loss. Otolaryngol. Clin. N. Am..

[47] Goldey E.S., Kehn L.S., Rehnberg G.L., Crofton K.M. (1995). Developmental exposure to polychlorinated-biphenyls (Aroclor-1254) reduces circulating thyroid-hormone concentrations and causes hearing deficits in rats. Toxicol. Appl. Pharmacol..

[48] Herr D.W., Goldey E.S., Crofton K.M. (1996). Developmental exposure to Aroclor 1254 produces low-frequency alterations in adult rat brainstem auditory evoked responses. Fundam. Appl. Toxicol..

[49] Crofton K., Ding D., Padich R., Taylor M., Henderson D. (2000). Hearing loss following exposure during development to polychlorinated biphenyls: A cochlear site of action. Hear. Res..

[50] Noorbakhsh S., Memari F., Farhadi M., Tabatabaei A. (2008). Sensorineural hearing loss due to *Toxoplasma gondii* in children: A case-control study. Clin. Otolaryngol..

[51] De Castro Corrêa C., Maximino L.P., Weber S.A.T. (2018). Hearing disorders in congenital toxoplasmosis: A literature review. Int. Arch. Otorhinolaryngol..

[52] Salviz M., Montoya J.G., Nadol J.B., Santos F. (2013). Otopathology in congenital toxoplasmosis. Otol. Neurotol..

[53] Cohen B.E., Durstenfeld A., Roehm P.C. (2014). Viral causes of hearing loss: A review for hearing health professionals. Trends Hear..

[54] Sheridan M.D. (1964). Final report of a prospective study of children whose mothers had Rubella in early pregnancy. Br. Med. J..

[55] Chandrasekhar S.S., Connelly P.E., Brahmbhatt S.S., Shah C.S., Kloser P.C., Baredes S. (2000). Otologic and audiologic evaluation of human immunodeficiency virus-infected patients. Am. J. Otolaryngol..

[56] Cureoglu S., Schachern P.A., Paparella M.M., Lindgren B.R. (2004). Cochlear changes in chronic otitis media. Laryngoscope.

[57] Houser D.S., Finneran J.J. (2006). Variation in the hearing sensitivity of a dolphin population determined through the use of evoked potential audiometry. J. Acoust. Soc. Am..

[58] Houser D.S., Gomez-Rubio A., Finneran J.J. (2008). Evoked potential audiometry of 13 Pacific bottlenose dolphins (*Tursiops truncatus*
*gilli*). Mar. Mamm. Sci..

[59] Sun J.J., Wang J.B., Wei N.R. (1987). Histopathological observation on the inner ear barotrauma in guinea pig. J. Tongji Univ. Nat. Sci..

[60] Wang J., Puel J.L. (2018). Toward cochlear therapies. Physiol. Rev..

[61] Jepson P.D., Bennett P.M., Deaville R., Allchin C.R., Baker J.R., Law R.J. (2005). Relationships between polychlorinated biphenyls and health status in harbor porpoises (*Phocoena phocoena*) stranded in the United Kingdom. Environ. Toxicol. Chem..

[62] Kannan K., Blankenship A., Jones P., Giesy J. (2000). Toxicity reference values for the toxic effects of polychlorinated biphenyls to aquatic mammals. Hum. Ecol. Risk Assess..

[63] Mancini P., Atturo F., Di Mario A., Portanova G., Ralli M., De Virgilio A., de Vincentiis M., Greco A. (2018). Hearing loss in autoimmune disorders: Prevalence and therapeutic options. Autoimmun. Rev..

[64] Davis H., Morgan C.T., Hawkins J.E., Galambos R., Smith F.W. (1950). Temporary deafness following exposure to loud tones and noise. Acta Oto-Laryngol..

[65] Kastelein R.A., Schop J., Gransier R., Hoek L. (2014). Frequency of greatest temporary hearing threshold shift in harbor porpoises (*Phocoena phocoena*) depends on the noise level. J. Acoust. Soc. Am..

[66] Finneran J.J. (2015). Noise-induced hearing loss in marine mammals: A review of temporary threshold shift studies from 1996 to 2015. J. Acoust. Soc. Am..

[67] Reichmuth C., Sills J.M., Mulsow J., Ghoul A. (2019). Long-term evidence of noise-induced permanent threshold shift in a harbor seal (*Phoca vitulina*). J. Acoust. Soc. Am..

[68] Migaki G., Sawa T.R., Dubey J.P. (1990). Fatal disseminated toxoplasmosis in a spinner dolphin (*Stenella longirostris*). Vet. Pathol..

[69] Di Guardo G., Proietto U., Di Francesco C.E., Marsilio F., Zaccaroni A., Scaravelli D., Mignone W., Garibaldi F., Kennedy S., Forster F. (2010). Cerebral toxoplasmosis in striped dolphins (*Stenella coeruleoalba*) stranded along the Ligurian Sea coast of Italy. Vet. Pathol..

[70] Mazzariol S., Marcer F., Mignone W., Serracca L., Goria M., Marsili L., Di Guardo G., Casalone C. (2012). Dolphin Morbillivirus and *Toxoplasma gondii* coinfection in a Mediterranean fin whale (*Balaenoptera physalus*). BMC Vet. Res..

[71] St Leger J., Raverty S., Mena A. (2018). Cetacea. Pathology of Wildlife and Zoo Animals.

[72] Yan J., Huang B., Liu G., Wu B., Huang S., Zheng H., Shen J., Lun Z., Wang Y., Lu F. (2013). Meta-analysis of prevention and treatment of toxoplasmic encephalitis in HIV-infected patients. Acta Trop..

[73] Peng H.P., Tan F., Lindsay D.S., Singh S.K. (2016). Pathogenesis of *Toxoplasma gondii* in Humans. Human Emerging and Re-Emerging Infections: Viral and Parasitic Infections.

[74] Van Bressem M.F., Raga J.A., Di Guardo G., Jepson P.D., Duignan P.J., Siebert U., Barrett T., de Oliveira Santos M.C., Moreno I.B., Siciliano S. (2009). Emerging infectious diseases in cetaceans worldwide and the possible role of environmental stressors. Dis. Aquat. Organ..

[75] Di Guardo G., Mazzariol S. (2013). *Toxoplasma gondii*: Clues from stranded dolphins. Vet. Pathol..

[76] Wang L., Chen H., Liu D., Huo X., Gao J., Song X., Xu X., Huang K., Liu W., Wang Y. (2013). Genotypes and mouse virulence of *Toxoplasma gondii* isolates from animals and humans in China. PLoS ONE.

[77] Di Guardo G., Centelleghe C., Mazzariol S. (2018). Cetacean host-pathogen interaction(s): Critical knowledge gaps. Front. Immunol..

[78] Ten Doeschate M.T., IJsseldijk L.L., Hiemstra S., De Jong E.A., Strijkstra A., Gröne A., Begeman L. (2017). Quantifying parasite presence in relation to biological parameters of harbour porpoises *Phocoena phocoena* stranded on the Dutch coast. Dis. Aquat. Organ..

[79] Siebert U., Wunschmann A., Weiss R., Frank H., Benke H., Frese K. (2001). Post-mortem findings in harbour porpoises (*Phocoena phocoena*) from the German North and Baltic Seas. J. Comp. Pathol..

[80] Siebert U., Pawliczkac I., Benke H., von Vietinghoff V., Wolf P., Pilāts V., Kesselring T., Lehnert K., Prenger-Berninghoff E., Galatius A. (2020). Health assessment of harbour porpoises (*Phocoena phocoena*) from Baltic area of Denmark, Germany, Poland and Latvia. Environ. Int..

[81] Jauniaux T., Petitjean D., Brenez C., Borrens M., Brosens L., Haelters J., Tavernier T., Coignoul F. (2002). Post-mortem findings and causes of death of harbour porpoises (*Phocoena phocoena*) stranded from 1990 to 2000 along the coastlines of Belgium and Northern France. J. Comp. Pathol..

[82] Jepson P.D., Baker J.R., Kuiken T., Simpson V.R., Kennedy S., Bennett P.M. (2000). Pulmonary pathology of harbour porpoises stranded in England and Wales between 1990 and 1996. Vet. Rec..

[83] Beineke A., Siebert U., MacLachlan M., Bruhn R., Thron K., Failing K., Müller G., Baumgärtner W. (2005). Investigations of the potential influence of environmental contaminants on the thymus and spleen of harbor porpoises (*Phocoena phocoena*). Environ. Sci. Technol..

[84] Desforges J.-W., Sonne C., Levin M., Siebert U., De Guise S., Dietz R. (2015). Immunotoxic effects of environmental pollutants in marine mammals. Environ. Int..

[85] Jepson P.D., Deaville R., Barber J.L., Aguilar A., Borrell A., Murphy S., Barry J., Brownlow A., Barnett J., Berrow S. (2016). PCB pollution continues to impact populations of orcas and other dolphins in European waters. Sci. Rep..

[86] Møhl B., Andersen S. (1973). Echolocation: High-frequency component in click of harbor porpoise *(Phocoena ph.* L.). J. Acoust. Soc. Am..

[87] Villadsgaard A., Wahlberg M., Tougaard J. (2007). Echolocation signals of wild harbour porpoises, *Phocoena phocoena*. J. Exp. Biol..

[88] Miller L.E., Wahlberg M. (2013). Echolocation by the harbor porpoise: Life in coastal waters. Front. Physiol..

[89] Kastelein R.A., Helder-Hoek L., Van de Voorde S. (2017). Hearing thresholds of a male and a female harbor porpoise (*Phocoena phocoena*). J. Acoust. Soc. Am..

[90] Ketten D.R., Popper A.N., Hawkins A. (2012). Marine Mammal Auditory System Noise Impacts: Evidence and Incidence. The Effects of Noise on Aquatic Life. Advances in Experimental Medicine and Biology.

